# What Becomes of the Microbes?

**Published:** 1896-04

**Authors:** 


### What Becomes of the Microbes?

The old question, "Where do all the pins go?" would seem easy of solution beside the same inquiry with regard to the germs, beneficent or disease-producing, that grow and multiply by myriads in soil, air and water. *The Hospital* gives us an answer, so far as those are concerned that effect an entrance into our systems—no small number, as will be seen from the following quoted paragraphs. The sum and substance of it all is that if the bodily health is good, the bacteria perish in the digestive organs, otherwise—so much the worse for us; and from these facts an enirely obvious moral is drawn. Says the article referred to:

"We hear so much of microbes, and are so constantly assured that the air is so full of them, that it becomes a matter of no small interest to ascertain how we are protected from them, or, it other words, how it is that living as we do in the very midst of a cloud of micro-organisms, which we know by experience are able very quickly to reduce to putrescence substances which, so far as chemical composition goes, are like unto ourselves, we still remain protected from their attacks. The vulnerable point clearly is the mucous membrane of the air-passages and the digestive organs. As regards the latter, we may well believe that in health we are protected by the activity of our digestive processes; but in reference to the air-ducts, over the moist surfaces of which the foulest air is constantly drawn, it is a problem of the greatest interest to decide whereabouts the microbes, which we know are constantly entering, are stopped. The recent researches of Dr. St. Clair Thomson and Dr. Hewlett, of the Bacteriological Department of the British Institute of Preventive Medicine, throw much light upon this question. They say that, on an average, about 1,500 micro-organisms are inhaled into the nose every hour; while in London it must be a common event for 14,000 of them to enter during one hour's tranquil respiration. Expired air, however, is practically sterile, and it would seem that this purification is not, as some have imagined, performed in the air-tubes of the lungs, for it has been found by repeated observation that they have vanished before reaching the trachea, the mucus from which is sterile. Evidently they are caught in the nose, for on testing air from the naso-pharynx they were found to be practically all gone. Nevertheless, the mucus in the nose does not appear to be itself a germicide. It does not kill the microbes, but it prevents their developing; and as microbes are only harmful by their monstrous power of multiplication this is sufficient. Meanwhile they are rapidly swept on by the cilia toward the digestive tract, where, doubtless, they share the common fate. The moral of all this is—breathe through your nose and keep your digestive organs in good working order, then the microbes, pathogenic, saprophytic, or whatever they may be, will meet their doom."—*The Literary Digest.*

